# lncRNA-*ZFAS1* induces mitochondria-mediated apoptosis by causing cytosolic Ca^2+^ overload in myocardial infarction mice model

**DOI:** 10.1038/s41419-019-2136-6

**Published:** 2019-12-09

**Authors:** Lei Jiao, Mengmeng Li, Yingchun Shao, Yuanyuan Zhang, Manyu Gong, Xuewen Yang, Yanying Wang, Zhongyue Tan, Lihua Sun, Lina Xuan, Qi Yu, Yanru Li, Yuqiu Gao, Heng Liu, Honglin Xu, Xiaohan Li, Yong Zhang, Ying Zhang

**Affiliations:** Department of Pharmacology (State-Province Key Laboratories of Biomedicine-Pharmaceutics of China, Key Laboratory of Cardiovascular Medicine Research, Ministry of Education), College of Pharmacy, Harbin Medical University, Harbin, Heilongjiang 150081 P.R. China

**Keywords:** Non-coding RNAs, Cardiovascular diseases

## Abstract

Previously, we have identified *ZFAS1* as a potential new long non-coding RNA (lncRNA) biomarker of acute myocardial infarction (MI) and as a sarcoplasmic reticulum Ca^2+^-ATPase 2a (SERCA2a) inhibitor, causing intracellular Ca^2+^ overload and contractile dysfunction in a mouse model of MI. In the current study, we aimed to evaluate the effects of *ZFAS1* on the apoptosis of cardiomyocytes in the MI mouse model. Knockdown of endogenous *ZFAS1* by virus-mediated silencing shRNA or si*ZFAS1* partially abrogated the ischemia-induced apoptosis of cardiomyocytes. Overexpression of *ZFAS1* in normal cardiomyocytes reduced the cell viability, similar to that observed in hypoxia-treated cardiomyocytes. Moreover, *ZFAS1* cardiac-specific knock-in mice showed impaired cardiac function, adversely altered Ca^2+^ homeostasis, repressed expression and activities of SERCA2a, and increased apoptosis. At the subcellular level, *ZFAS1* induced mitochondrial swelling and showed a pronounced decrease in mitochondrial membrane potential. At the molecular level, *ZFAS1* activated the mitochondria apoptosis pathway, which could be nearly abolished by a calcium chelator. The effects of *ZFAS1* were readily reversible upon knockdown of this lncRNA. Notably, *ZFAS1-FD* (only functional domain) mimicked the effects of full-length *ZFAS1* in regulation of cardiomyocyte apoptosis. In conclusion, our study shows that *ZFAS1*, an endogenous SERCA2a inhibitor, induces mitochondria-mediated apoptosis via cytosolic Ca^2+^ overload. Therefore, anti-*ZFAS1* might be considered a new therapeutic strategy for protecting cardiomyocytes from MI-induced apoptosis.

## Introduction

Myocardial infarction (MI), a leading cause of heart failure, is a severe threat to human lives^1,2^. The pathogenesis of MI involves multiple conditions, with cardiomyocyte apoptosis being one of the most crucial components^3,4^. Intrinsic pathway (also called mitochondria-mediated apoptotic pathway) and extrinsic pathway are the two pathways that modulate apoptosis, with the former playing an important role in cardiomyocyte apoptosis^5,6^. Mitochondria-mediated apoptosis is initiated by intracellular stimuli, such as oxidative stress, hypoxia, and nutrient deprivation, which causes an imbalance in the expression of Bcl_2_ family proteins (up-regulated pro-apoptotic proteins (Bax) and downregulated anti-apoptotic proteins (Bcl_2_)), leading to the induction of mitochondrial outer membrane permeabilization (MOMP) and release of cytochrome C. Cytochrome C promotes the assembly of caspase-9-associated apoptosome. Subsequently, caspase-9 triggers the caspase cascade, leading to the activation of caspase-3/7 and finally apoptosis^7,8^. Recently, studies have confirmed that cytosolic Ca^2+^ overload could induce mitochondria-mediated apoptosis^9,10^. Yet, how cytosolic Ca^2+^ overload inducing cardiomyocytes apoptosis during MI remains poorly understood.

Long noncoding RNAs (lncRNAs), a newly discovered class of non-protein-coding RNAs, have been reported to be involved in multiple heart diseases^11–13^. Growing evidences had demonstrated that lncRNA-*ZFAS1*, an antisense lncRNA to the 5′ end of the protein-coding gene Znfx1, had a significant role in the regulation of tumor^14–16^. In our previous study, we identified *ZFAS1* as an independent predictor of acute MI (AMI)^17^. Additionally, we found that *ZFAS1* bound to and inhibited the intracellular level and activity of sarcoplasmic reticulum Ca^2+^-ATPase 2a (SERCA2a) protein, and contributed to the impairment of cardiac contractile function in MI^18^. Therefore, *ZFAS1* might be considered as a new therapeutic target for preserving cardiac function under pathological conditions of the heart. However, it is not yet clear whether *ZFAS1* is involved in mitochondria-mediated cardiomyocyte apoptosis via cytosolic Ca^2+^ overload.

In the current study, we aimed to further clarify the role of *ZFAS1* in mitochondria-mediated cardiomyocyte apoptosis by loss and gain of function approaches in MI mice model. The results of this study are expected to provide a basis for developing novel therapeutic strategies for protecting cardiomyocytes from MI-induced apoptosis.

## Materials and methods

### The mouse model of MI

A mouse model of MI was obtained by left anterior descending coronary artery (LAD) occlusion with C57BL/6 mice ranging from 8 to 10 weeks in age and weighing between 22 g and 25 g as described previously in detail^19^. Significant elevation of S-T segment in electrocardiograph (ECG) was observed in the MI group. The mice were sacrificed at 12 h after MI. The sacrificed mice were removed and randomization and blinding were adopted in animal experiments. Use of animals was approved by the Ethic Committees of Harbin Medical University.

### Cardiac-specific ZFAS1 knock-in mice

Cardiac-specific *ZFAS1* knock-in (TG) mice were generated by crossing *ZFAS1* flox/flox mice (Cyagen Biosciences Inc.) with C57BL/6 background and a-myosin heavy chain promoter–driven Cre mice (αMHC-Cre, Cyagen Biosciences Inc.) as described previously^20^.

### Echocardiographic assessment of cardiac function

The left ventricular internal dimension at end-diastole (LVIDd), left ventricular internal dimension at systole (LVIDs), and ejection fraction (EF) of mice models were assessed by an echocardiographic system (Visualsonics, Toronto, ON, Canada) as described previously^21^. The fractional shortening (FS) was calculated according to the equation: (LVIDd−LVIDs)/LVIDd × 100.

### Construction and delivery of viral vectors for ZFAS1 overexpression and knockdown

AAV9 vectors carrying a short RNA fragment for silencing *ZFAS1* (sh*ZFAS1*-V, “V” representing virus) or *ZFAS1* sequence (*ZFAS1*-V) were constructed (Lederer biological technology Co., Ltd., Guangzhou, Guangdong, China) as described previously^18^. C57BL/6 mice received the virus solution (2 × 10^11^ genome-containing particles (GC)/animal) via tail vein injection. The following experiments would be done after one-time injection of *ZFAS1*-V or sh*ZFAS1*-V for 4 weeks.

### Neonatal mouse cardiomyocytes (NMCMs) culture and treatment

Cardiomyocytes isolated from 1 to 3-day-old neonatal mice (C57BL/6) were deprived of serum and placed in an anoxic chamber (5% CO_2_ and 95% N_2_) for 12 h to mimic myocardial ischemia in vitro. The *ZFAS1*-specific siRNA (si*ZFAS1*), commercially synthesized by Ribobio (Guangzhou, Guangdong, China, sense: GCGUGAACUCCUGAGGCGAdTdT, antisense: UCGCCUCAGGAGUUCACGCdTdT) was transfected into cells for *ZFAS1* knockdown according to the manufacturer’s protocol. *ZFAS1* transcript cDNA, inserted into the pCDNA3.1 (pCDNA-*ZFAS1, ZFAS1-P*), were constructed and transfected into cells (2 mg/L) for *ZFAS1* overexpression. After transfected with pCDNA-*ZFAS1* for 24 h, BAPTA (1,2-bis(o-aminophenoxy)ethane-N,N,N′,N′-tetraacetic acid) (10 μmol/L) was added for Ca^2+^ chelation*. ZFAS1*-FD, synthesized by Lederer Biological Technology (Guangdong, China, 5′-UGCGUGCCAAGCGCGACAUGGCGCGGAAGCCGAGAAGCCCCGGAGGCCC-3′ was transfected into cells for *ZFAS1*-FD overexpression. Cyclopiazonic acid (CPA) was added to the NMCMs for SERCA2a inhibition at the concentration of 5 μmol/L. The NMCMs were collected for the following experiments after transfection for 48 h or 72 h.

### Quantitative real-time PCR (qPCR)

qPCR was performed as previously described^22^. The data were collected from three separate experiments. Sequences for qPCR primers: *ZFAS1* (mouse): forward 5′-AGCGTTTGCTTTGTTCCC-3′ and reverse 5′-CTCCCTCGATGCCCTTCT-3′; SERCA2a (mouse): forward 5′-TAAATGCCCGCTGTTTTGCT-3′ and reverse 5′-TTGTCATCTGCCAGGACCAT-3′; β-actin (mouse): forward 5′-ACTGCCGCATCCTCTTCCT-3′ and reverse 5′-TCAACGTCACACTTCATGATGGA-3′.

### MTT assay for cell viability

Cardiomyocytes were cultured in 96-well culture clusters (about 1*10^4^ per well), and then the cells were transfected with *ZFAS1-*specific siRNA or pCDNA-*ZFAS1* plasmid vectors for 48 h. The cells cultured in complete medium under a normoxic atmosphere were considered as blank control. Particularly, some cells need hypoxia treatments. The cells were incubated for 4 h in a medium containing 0.5% 3-[4,5-dimethylthiazol-2-yl]-2,5-diphenyl-tetrazolium bromide (MTT). The amount of blue formazan dye formed from MTT is proportional to the number of survival cells. The MTT reaction was terminated by adding DMSO to the medium followed by incubation for 10 min at room temperature. The absorbance was read at 490 nm in a spectrophotometer (BioTek, USA).

### TUNEL assay

Terminal deoxynucleotide transferase dUTP nick end labeling (TUNEL) staining was used to evaluate the apoptosis of cultured cardiomyocytes. Briefly, cardiac myocytes cultured on coverslips in 24-well plates were fixed in 4% paraformaldehyde. The TUNEL staining was done using the in situ cell death detection kit (Minneapolis, MN, USA) according to the manufacturer’s protocol. The numbers of TUNEL-positive cells and the total cells were counted under a confocal microscopy.

### Western blot

Proteins isolated from cells (40–60 μg) or myocardial tissue (80–120 μg) were separated by SDS–PAGE (10–15% polyacrylamide gels). Partitioned proteins were transferred to Pure Nitrocellulose Blotting Membrane (PALL, New York, USA). The membrane was probed with a primary antibodies for SERCA2a (#A1097; ABclonal, Wuhan, China), Caspase3 (Cell Signaling Technology® #9662S), Cleaved Caspase3 (Cell Signaling Technology® #9664S), Caspase9 (Cell Signaling Technology® #9504), Bax (Gene Tex® #GTX32465), Bcl_2_ (Gene Tex® #GTX100064), p-CAMKII (Affbiotech, #AF3493), CAMKII (Affbiotech, #AF6493), and Connexin43 (EMD Millipore, #AB1727) at 4 °C overnight. Then, the membranes were incubated with secondary antibodies (Jackson Immuno Research, West Grove, PA, USA). Blots were detected and analyzed with the Odyssey v1.2 software (LI-COR Biosciences, Lincoln, NE, USA) for each group and normalized to β-actin or β-Tubulin band intensity. The final results were expressed as fold changes and the experiment was performed in triplicate and repeated three times.

### LIVE/DEAD viability/cytotoxicity kit stains

The cultured NMCMs were seeded in six-well plates and the components of the LIVE/DEAD Viability/Cytotoxicity Kit (Cat. L3224, Life technologies, USA) were added to each well. Cells with compromised membranes exhibit red-fluorescence from the live-cell-impermeant nucleic acid stain ethidium homodimer-1. Cells with intact cell membranes could convert nonfluorescent calcein AM into bright green-fluorescent calcein using nonspecific cytosolic esterases. Images were observed by confocal laser microscope (Olympus, Tokyo, Japan).

### Assessment of mitochondrial membrane potential

JC-1 (5,5′,6,6′-tetrachloro-1,1′,3,3′-tetraethylbenzimidazolcarbocyanine iodide) has been extensively used to study the loss of the mitochondrial membrane potential which occurs during apoptosis. In normal cells, due to high membrane potential, the dye concentrates in the mitochondrial matrix, and it forms red-fluorescent aggregates (J-aggregates). Any event that dissipates the mitochondrial membrane potential prevents the accumulation of the JC-1 dye in the mitochondria and thus, the dye is dispersed throughout the entire cell leading to a shift from red (J-aggregates) to green fluorescence (JC-1 monomers). A decrease in red/green ratio is indicative of apoptosis. The cells were grown in 24-well plate and transfected with *ZFAS1*-siRNA before hypoxia treating for 12 h. The treated cells were washed with PBS and stained with 2 mg/ml of JC-1 dye in DMEM media at 37 °C in dark for 20 min. Laser confocal microscopy was adopted for observation and photo taking.

### Flow cytometry

Apoptosis was detected by measuring phosphatidyl serine exposure and membrane permeability of cells. Cardiomyocytes were harvested and double-stained with FITC-conjugated Annexin V and propidium iodide (PI) (absin Annexin V/FITC Apoptosis Detection Kit). Samples were analyzed by the FACScan flow cytometer with Cell Quest software (Beckman Coulter, USA).

### Optical mapping

The real-time changes of intracellular Ca^2+^ concentration ([Ca^2+^]_i_) were measured by optical mapping system (MICAM05, Brainvision, Tokyo, Japan). The optical mapping was performed according to the procedures described previously^23^. An image-capturing software (BV_MC05E; Brainvision, Tokyo, Japan) was adopted for optical recording of the heart at 6 Hz field stimulation and an image analysis software (BV-Analyze; Brainvision) was used for data analysis.

### Measurement of SERCA2a activity

The SERCA2a activity was determined by Ca^2+^ ATPase assay kit (Nanjing Jiancheng Bioengineering Institute, Nanjing, China) according to the manufacturer’s protocol. Briefly, cardiac tissue or NMCMs were treated as specified. The amount of inorganic phosphate liberated from ATP hydrolysis was used to evaluate the activity of SERCA2a.

### Immunofluorescence staining

Immunofluorescence staining was performed on cardiac tissue to determine Cx43 expression. The heart tissue was paraffin-embedded and sectioned for following staining. After dewaxing with xylene and antigen repair, the sectioned tissue was blocked with 10% goat serum overnight and incubated with anti-Cx43 (1:2000), alpha actin (1:300) overnight at 4 °C and then with the conjugated secondary antibody for 1 h. The nuclei were visualized with DAPI (4′,6-diamidino-2-phenylindole) at room temperature for 30 min, and images (×40 magnification) were captured by a confocal fluorescent microscope.

### Isolation of adult mouse cardiomyocytes

Cardiomyocytes were isolated from adult *ZFAS1* knock-in mice or WT as described previously^18^. Briefly, adult mice were anesthetized and the hearts were rapidly excised. The aorta of the heart was cannulated on a constant-flow Langendorff apparatus. After digestion, the heart tissue had become softened and gently minced into small chunks, which were then equilibrated in Tyrode’s solution with 200 μM CaCl_2_ and 1% bovine serum albumin at room temperature.

### Data analysis

Pooled data are presented as mean ± SEM values and analyzed with SPSS 13.0 software. Statistical comparisons among multiple groups were performed using analysis of variance (ANOVA) followed by Dunnett’s test. Student's *t*-test was carried out for comparisons between two groups. A two-tailed *P* < 0.05 was taken to indicate a statistically significant difference.

## Results

### Knockdown of ZFAS1 protects cardiomyocytes against MI

**Fig. 1:**
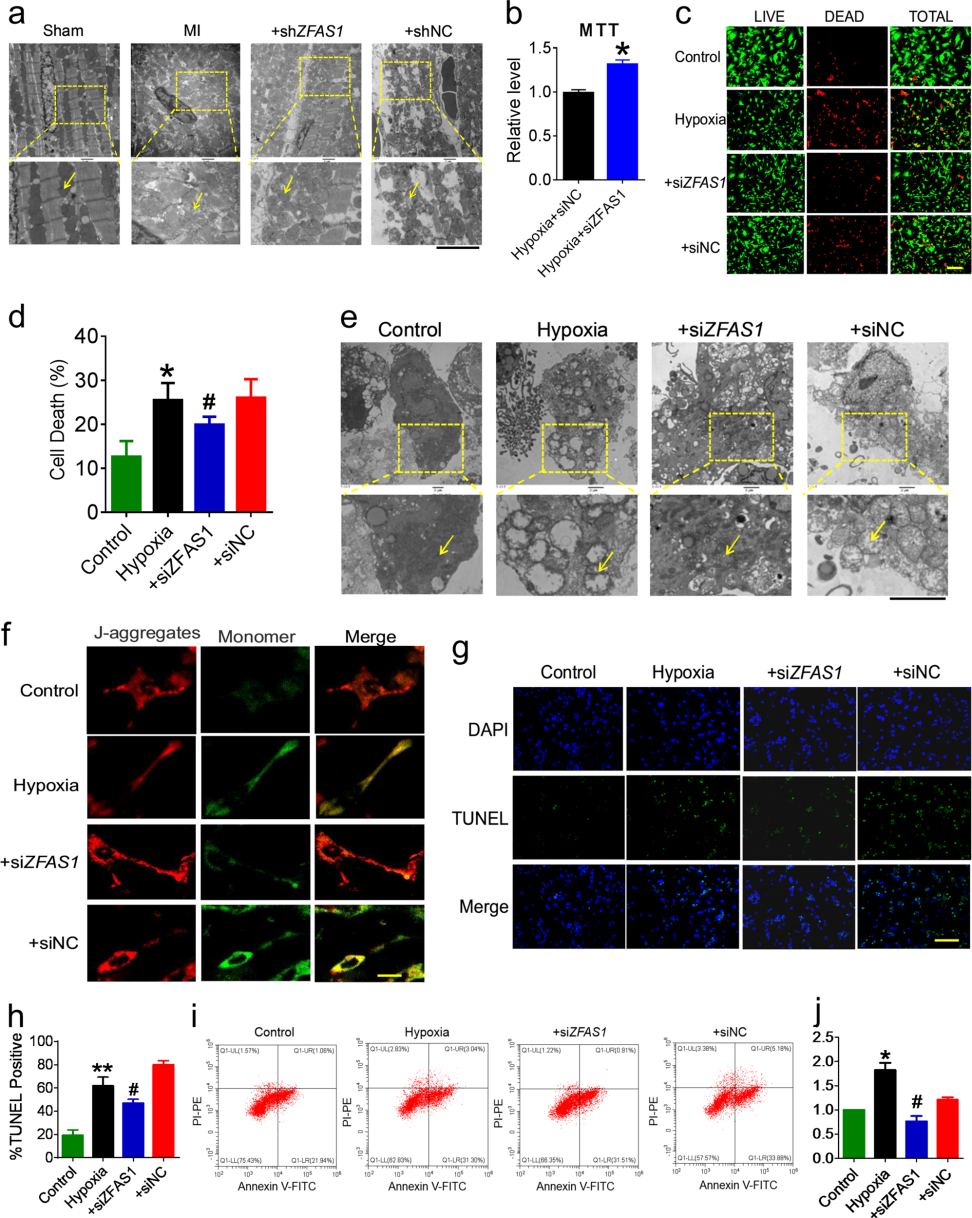
*ZFAS1* knockdown inhibits cardiomyocyte apoptosis caused by myocardial infarction. **a** Effects of *ZFAS1* knockdown on the mitochondrion in the myocardium of MI mice, observed by electron microscope. Yellow arrows pointing to the mitochondrion. Images are presented with a magnification of ×10,000 for the top and ×40,000 for the down panel. It was repeated for three times with similar results. **b** MTT assay showing that *ZFAS1* knockdown increased the cell viability of hypoxia-treated cells. *P < 0.05 vs. Hypoxia + siNC, *n* = 13. **c**, **d** LIVE/DEAD Viability/Cytotoxicity Kit stains were used to detect the effects of *ZFAS1* on cardiomyocytes. Cells with compromised membranes exhibit red-fluorescence, while those with intact cell membranes show bright green-fluorescence. *P < 0.05 vs. control group, *n* = 5; ^#^*P* *<* 0.05 vs. hypoxia group, *n* = 5. Magnification, ×200. **e** Effects of *ZFAS1* knockdown on the mitochondrion in hypoxia-treated cardiomyocytes, visualized by electron microscope (magnification, ×10,000 for the top and ×40,000 for the down panel). Similar results were consistently observed in another two batches of cells. **f** *ZFAS1* knockdown improved the mitochondrial membrane potential, as observed by JC-1 staining. The red-fluorescent aggregates (J-aggregates) represent high membrane potential; green fluorescence (JC-1 monomers) represents dissipated mitochondrial membrane potential. Magnification, ×1200. Similar results were consistently observed in another two batches of cells. **g**, **h** The influence on apoptosis with *ZFAS1* knockdown was examined via TUNEL assay. Blue, DAPI staining for nucleus; green, TUNEL-positive staining for apoptotic cells. **P < 0.01 vs. control group, ^#^*P* < 0.05 vs. hypoxia group, the results are expressed as the means ± SEM of four independent experiments. Magnification, ×200. **i, j** Cells were harvested and processed for apoptosis assay using the Annexin V-FITC Apoptosis Detection Kit by flow cytometry. *P < 0.05 vs. control group, ^#^*P* < 0.05 vs. hypoxia group, the results are expressed as the means ± SEM of four independent experiments.

Recently, we demonstrated that *ZFAS1* expression is markedly elevated in the myocardium of mice with AMI (12 h post-MI)^18^. Here, we first established a MI model and confirmed that the cardiac function of the MI mice was decreased obviously (Fig. S1). Furthermore, mitochondria turgescence and fragmented cristae were observed in the heart tissue from MI mice (Fig. 1a). Next, we verified the expression level of *ZFAS1* in the cardiac tissue of MI mice and hypoxia-treated NMCMs. Compared with the Sham or Control group, the expression level of *ZFAS1* was increased (Fig. S2). We then employed a loss-of-function strategy with adeno-associated virus serotype 9 (AAV9) vector carrying a *ZFAS1*-shRNA fragment (sh*ZFAS1*-V) and investigated if knocking down *ZFAS1* could alter pathological conditions of the heart. The efficiency of sh*ZFAS1*-V for *ZFAS1* knockdown was verified by qRT-PCR (Fig. S3a). In the current study, the mitochondrial swelling and myofibril breakage caused by MI in the cardiac tissue were nearly abolished by sh*ZFAS1*-V, as evident under electron microscope (Fig. 1a). In vitro, si*ZFAS1* increased the viability of NMCMs exposed to hypoxic environment (Fig. 1b). The efficiency of si*ZFAS1* for *ZFAS1* knockdown both in normal and hypoxia-treated NMCMs was verified by qRT-PCR (Fig. S3b, c). Using LIVE/DEAD Viability/Cytotoxicity Kit that stains cells with compromised membranes to show red-fluorescence and cells with intact cell membranes to show bright green-fluorescence, we observed that the hypoxia-treated cardiac myocytes exhibited more number of cells showing red-fluorescence than the control group; however, hypoxia-treated cells transfected with si*ZFAS1* showed an obvious reduction of red-fluorescence. It indicated that *ZFAS1* knockdown could restore the viability of hypoxia-treated cardiomyocytes (Fig. 1c, d). Moreover, the hypoxia treatment induced mitochondria turgescence and fragmented cristae in NMCMs were rescued by *ZFAS1* knockdown (Fig. 1e). To further assess the influence of *ZFAS1* on mitochondria, the JC-1 assay was done to evaluate the loss of mitochondrial membrane potential, which occurs during apoptosis. The results showed that compared with the control group, the mitochondrial membrane potential of hypoxia-treated NMCMs were notably compromised, presenting as a large area of green fluorescence. However, the si*ZFAS1*-transfected cells exhibited reduced green fluorescence after 12 h of hypoxia treatment compared with the normal control group (Fig. 1f). These results indicated that cells transfected with si*ZFAS1* might be resistant to hypoxia-induced apoptosis of cardiomyocytes. To clarify this, we investigated the effects of *ZFAS1* on apoptosis of cardiomyocytes induced by hypoxia treatment. As shown in Fig. 1g, h, the number of TUNEL-positive cells was significantly decreased after transfection with si*ZFAS1*, whereas siNC did not elicit any significant changes. Apoptosis was determined through PI Annexin V double staining and FACS analysis. As shown in Fig. 1i, j, the percentage of cells stained with Annexin V was increased after hypoxia for 12 h, while it was significantly reduced in case of cells transfected with si*ZFAS1*. These data demonstrated that knockdown of *ZFAS1* protects cardiomyocytes against apoptosis.

**Fig. 2:**
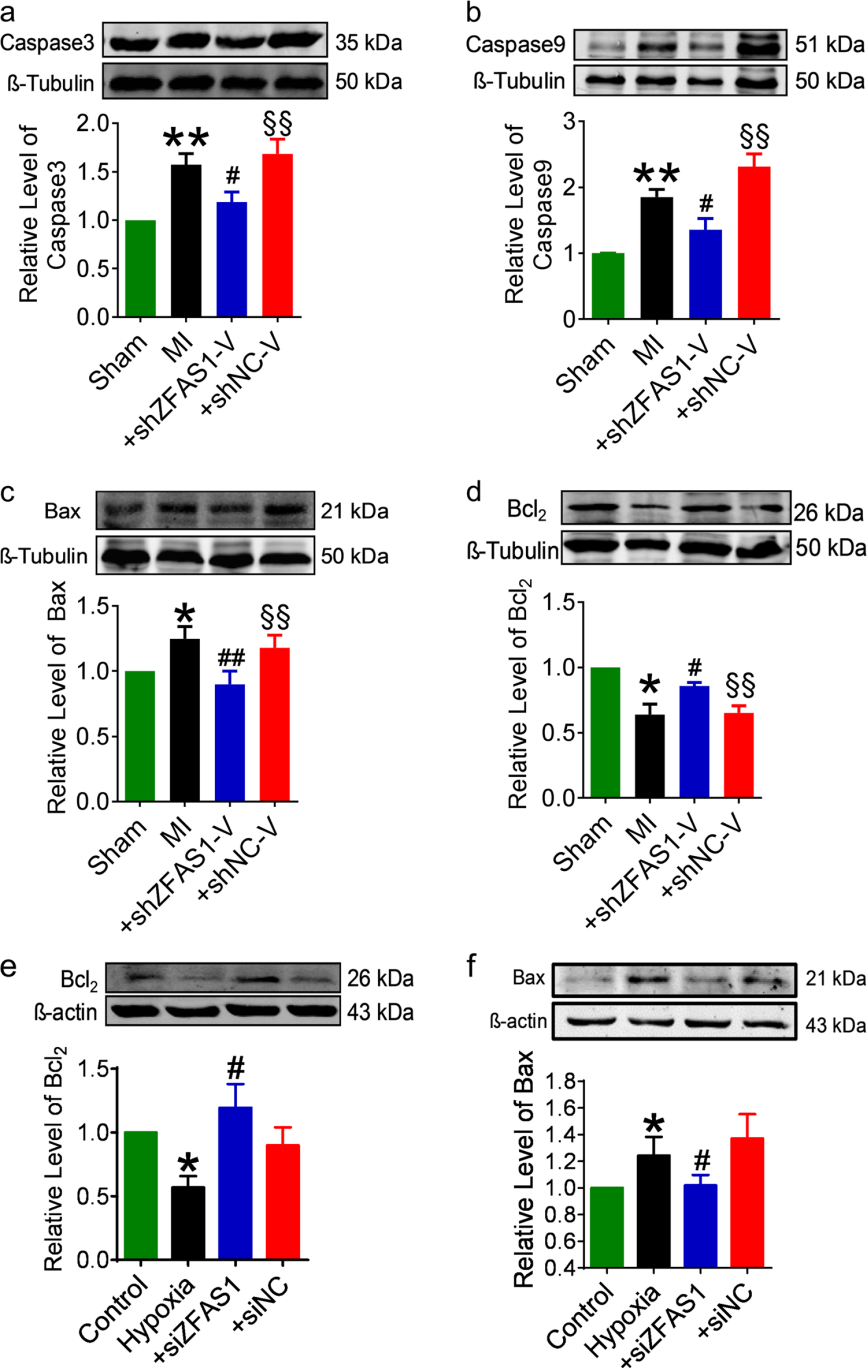
Effects of knocking down *ZFAS1* on the expression of apoptosis related proteins. **a**–**d** The expression of caspase-3, caspase-9, and Bax was significantly increased, and that of Bcl_2_ was decreased in heart with myocardial infarction (MI) compared with Sham-operation control mice. The expression was reversed by transfection with AAV9-sh*ZFAS1*. *P < 0.05, **P *<* 0.01 vs. Sham, ^#^*P* < 0.05 vs. MI, ^§§^*P* < 0.01 vs. MI + sh*ZFAS1*-V; *n* = 5 for caspase-3, caspase-9, and Bcl_2_, *n* = 7 for Bax. **e**, **f** The expression of Bax was significantly increased and Bcl_2_ was decreased in cultured cardiomyocytes after hypoxia treatment for 12 h. Note that silencing *ZFAS1* by si*ZFAS1* normalized the Bax and Bcl_2_ expression.*P *<* 0.05 vs. Control, ^#^*P* *<* 0.05 vs. Hypoxia; *n* = 6 for Bax, *n* = 5 for Bcl_2._ Data are presented as means ± SEM.

Next, we checked the expression of apoptosis-associated proteins from the mitochondria-mediated apoptotic pathway. As shown in Fig. 2a–d, the cardiac expression of caspase-3, caspase-9, and Bax proteins were prominently increased in MI mice and Bcl_2_ was decreased relative to the sham animals. Notably, sh*ZFAS1*-V reversed the abnormal expression of these proteins. Similar patterns of expression alterations of Bax and Bcl_2_ proteins were consistently observed in NMCMs cultured under hypoxic insult and with treatments with varying constructs (Fig. 2e, f). In all cases, the negative control construct shNC-V and siNC did not affect the deleterious alterations in MI mice and hypoxia-treated NMCMs.

### Upregulation of ZFAS1 expression induces cardiomyocyte apoptosis

**Fig. 3:**
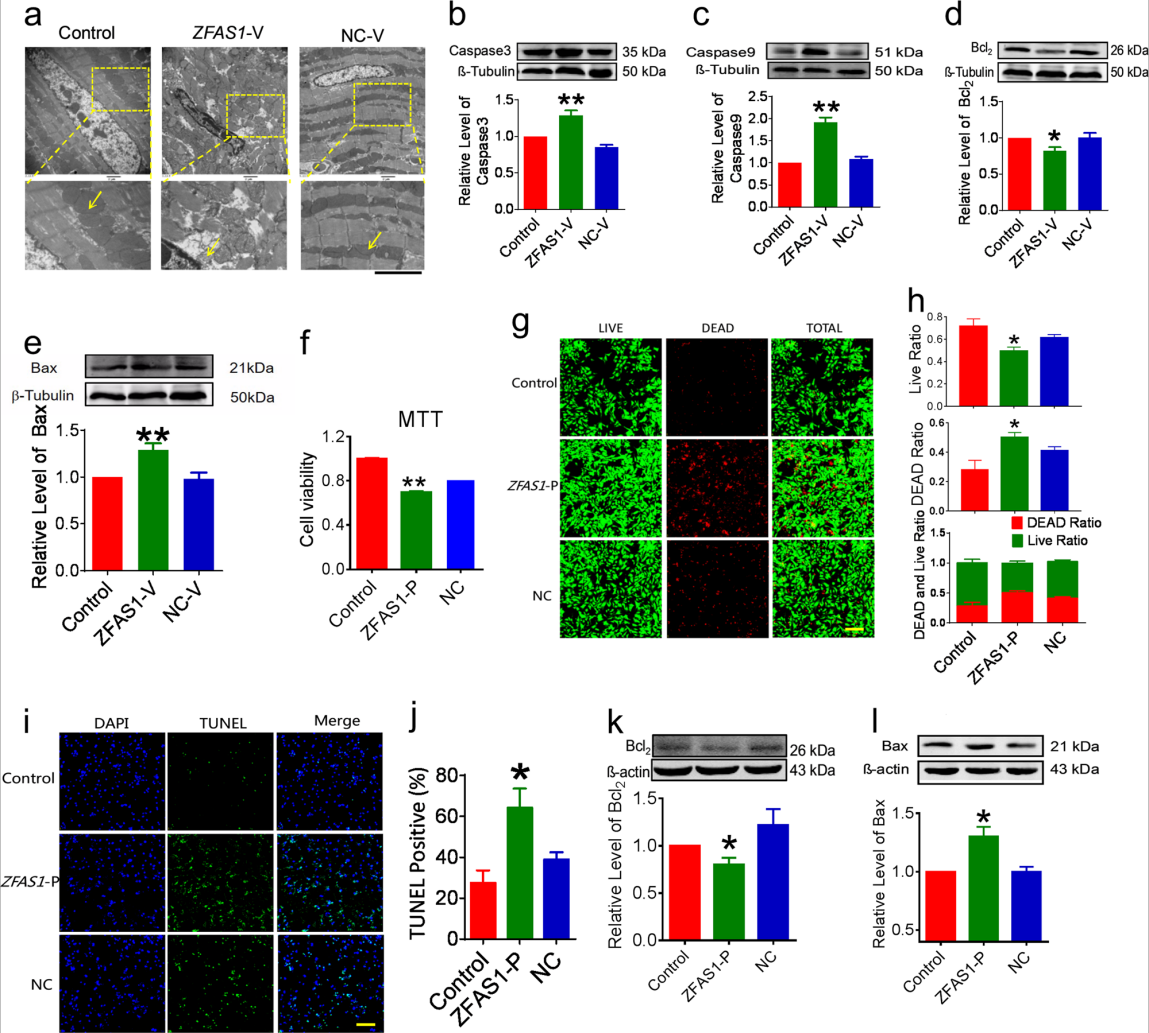
*ZFAS1* overexpression in healthy mice induces apoptosis. **a** Electron microscopy images showing the effects of *ZFAS1* overexpression on the mitochondria in the myocardium of mice (magnification, ×10,000 for the top and ×40,000 for the down panel). **b**–**e** The expression of apoptosis-related proteins including caspase-3, caspase-9, Bcl_2_, and Bax were quantified by western blot. *P < 0.05, **P < 0.01 vs. control group, *n* = 6 for caspase-3 and caspase-9, *n* = 5 for Bcl_2_ and Bax. **f** MTT assay to evaluate the cell viability of *ZFAS1*-overexpressing cardiacmyocytes generated by pCDNA-*ZFAS1* vector (*ZFAS1*-P) transfection in nonhypoxia cardiomyocytes. **P < 0.01 vs. Control Group, *n* = 13. **g**, **h** LIVE/DEAD Viability/Cytotoxicity Kit stains were used to detect the effect of *ZFAS1* overexpression on the viability of cardiacmyocytes. *P < 0.05 vs. Control Group, *n* = 7. Magnification, ×200. **i**, **j** The effect of *ZFAS1* on cardiomyocyte apoptosis was validated by TUNEL assay. Blue, DAPI staining for nucleus; green, TUNEL-positive staining for apoptotic cells. *P < 0.05 vs. control group, the results are expressed as the means ± SEM of four independent experiments. Magnification, ×200. **k**, **l** The expression of Bcl_2_ was significantly decreased and Bax was increased in cultured cardiomyocytes after *ZFAS1* overexpression. *P *<* 0.05 vs. control group, *n* = 5. Data are presented as means ± SEM.

The data presented above suggested that knockdown of *ZFAS1* protects myocardium against MI-induced cardiomyocytes apoptosis. If this is true, overexpression of *ZFAS1* in otherwise normal mice and NMCMs should be able to reproduce the phenotypes of MI and hypoxia-induced apoptosis. To test this hypothesis, we utilized the gain-of-function approach for our subsequent experiments. The AAV9 vector carrying the *ZFAS1* gene (*ZFAS1*-V) was constructed for its overexpression under in vivo conditions and the pCDNA-*ZFAS1* vector (*ZFAS1*-P) was transfected to NMCMs for overexpression in vitro conditions. The efficiency of *ZFAS1*-V and *ZFAS1*-P for *ZFAS1* overexpression were verified by qRT-PCR (Fig. S4). In sharp contrast to *ZFAS1* silencing, forced expression of *ZFAS1* with *ZFAS1*-V by tail vein injection caused mitochondrial swelling, as observed under electron microscope (Fig. 3a), and elevated expression of caspase-3, caspase-9, and Bax, and decreased expression of Bcl_2_ at the protein level (Fig. 3b–e). At the cellular level, *ZFAS1*-P-transfected NMCMs exhibited impaired cell viability (Fig. 3f–h). Moreover, TUNEL assay demonstrated that *ZFAS1* overexpression caused cardiomyocyte apoptosis (Fig. 3i, j). At the subcellular level, *ZFAS1*-P-transfected NMCMs showed downregulated Bcl_2_ protein and upregulated expression of Bax protein (Fig. 3k, l).

### Downregulation and dysfunction of SERCA2a induce cardiomyocyte apoptosis in ZFAS1 transgenic mice (TG)

**Fig. 4:**
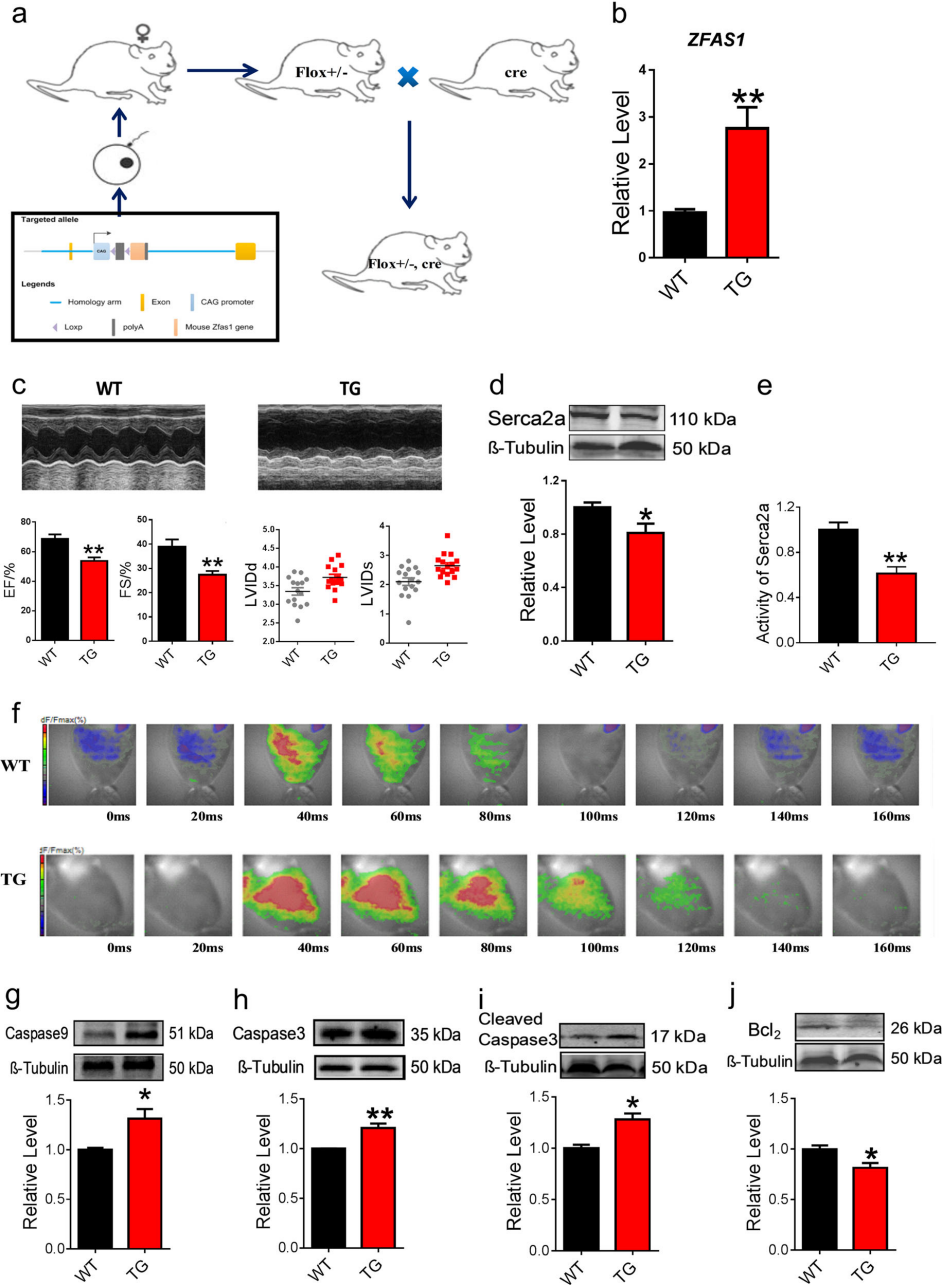
Impairment of cardiac function and increased apoptosis in *ZFAS1* transgenic heart (TG). **a** Schematic diagram of the generation of cardiac-specific *ZFAS1* knock-in mice. **b** Verification of the expression of *ZFAS1* in the heart tissue of *ZFAS1* knock-in mice (TG).**P < 0.01 vs. wide type (WT), *n* = 14. **c** The cardiac function of *ZFAS1* TG mice. *ZFAS1* TG mice showed decreased ejection fraction (EF) and fractional shortening (FS), and enlarged left ventricular internal dimension at end-diastole (LVIDd) and left ventricular internal dimension at systole (LVIDs).**P *<* 0.01 vs. WT, *n* = 12. **d** Downregulation of SERCA2a expression in *ZFAS1* knock-in mice at protein levels.*P *<* 0.05 vs. WT, *n* = 7. **e** Downregulation of SERCA2a activity in *ZFAS1* knock-in mice.**P *<* 0.01 vs. WT, *n* = 5. **f** Impairment of intracellular Ca^2+^ homeostasis by *ZFAS1*, assessed by optical-mapping techniques in *ZFAS1* knock-in mice. **g**–**j** Western-blot analysis of the expression of apoptosis-related proteins in TG mice.*P < 0.05,**P < 0.01 vs. WT, *n* = 5 for caspase-9, cleaved caspase-3, and Bcl_2_,*n* = 3 for caspase-3. Data are presented as means ± SEM.

To further investigate the role of *ZFAS1* in MI, *ZFAS1* cardiac-specific knock-in (TG) mice were constructed (Fig. 4a). The expression of *ZFAS1* in the cardiac tissue of the TG was verified by qRT-PCR (Fig. 4b). As depicted in Fig. 4c, TG showed significantly reduced EF and FS, and enlarged LVIDd and LVIDs. Similar to our previous study, *ZFAS1* knock-in mice exhibited decreased expression of SERCA2a both at the protein and mRNA levels (Fig. 4d, Fig. S5). Additionally, TG showed impaired activity of SERCA2a in the cardiac tissue (Fig. 4e). Moreover, we evaluated the effects of *ZFAS1* on the expression of other proteins that can modify SERCA activity or regulate cardiomyocyte–cardiomyocyte coupling. As shown in Fig. S6, *ZFAS1* had no obvious effects on the expression of P-CaMKII, CaMKII, or Cx43 protein levels. The calcium homeostasis of the TG was also assessed by optical-mapping techniques. Intriguingly, as depicted in Fig. 4f, the decaying phase time due to Ca^2+^ was significantly delayed in the TG compared with the wide type mice (WT). The resting intracellular Ca^2+^ in the cardiomyocytes isolated from TG was significantly increased comparing with WT (Fig. S7). Additionally, compared with the WT group, the expression of apoptosis-associated proteins—caspase-9, caspase-3, cleaved caspase-3—were significantly increased and Bcl_2_ was decreased in the cardiac tissue of TG (Fig. 4g–j). Collectively, downregulation and dysfunction of SERCA2a induced cardiomyocytes apoptosis in *ZFAS1-TG*.

CPA, an inhibitor of SERCA2a, could induce the dysfunction of SERCA2a^24^. Our expriments confirmed that CPA could inhibit the activity of SERCA2a and the inhibition of SERCA2a did not affect the expression of *ZFAS1* (Fig. S8). Conversely, *ZFAS1* exhibited time-dependent inhibition effects on SERCA2a activities (Fig. S9). Furthermore, CPA could induce the resting calcium overload in the cardiomyocytes (Fig. S10a) and cause impairment of the protective effects of si*ZFAS1* on calcium homeostasis in hypoxia-treated cardiomyocytes (Fig. S10b).

### ZFAS1-induced cytosolic Ca^2+^ overload causes cardiomyocyte apoptosis

**Fig. 5:**
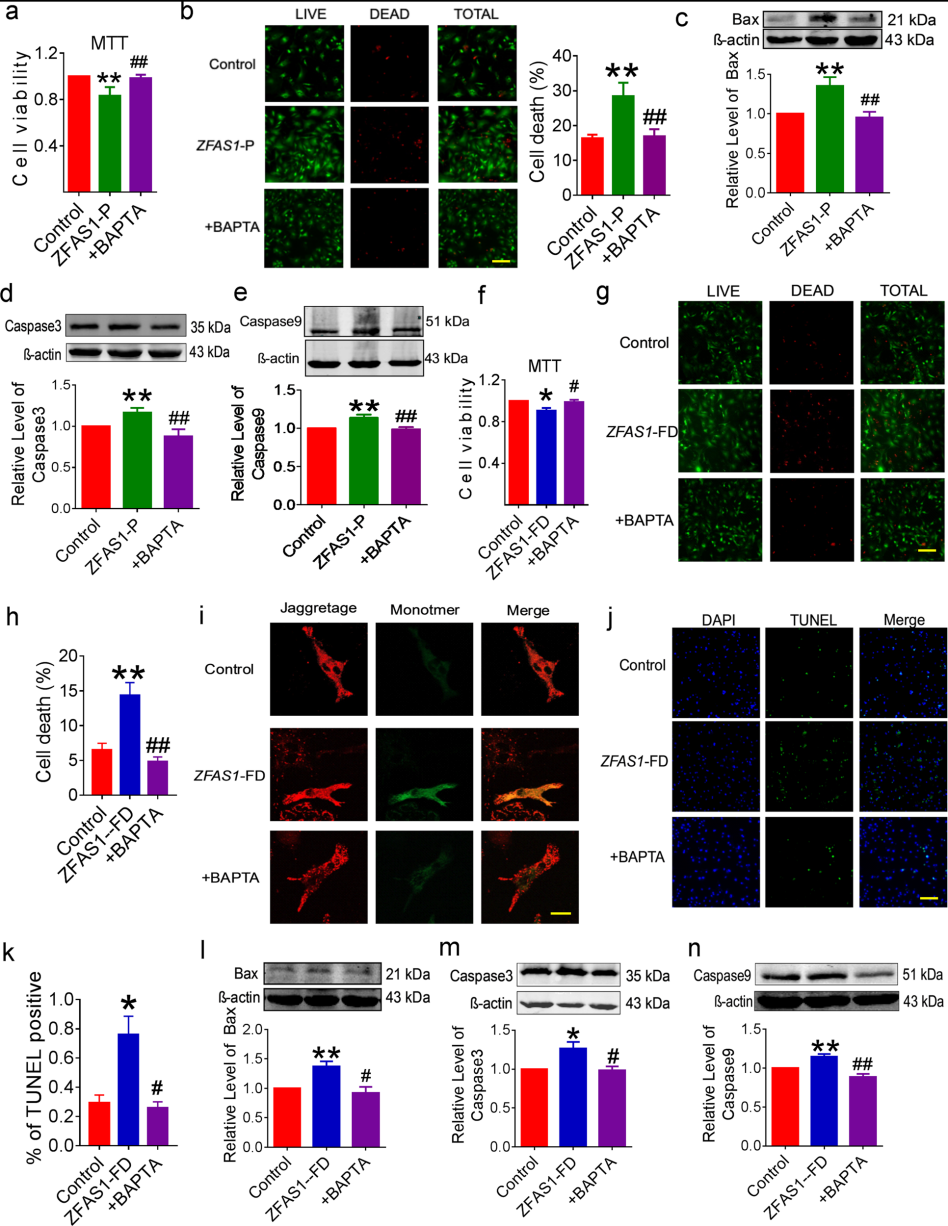
Calcium chelator *BAPTA* abolishes apoptosis induced by *ZFAS1* in cardiomyocytes. **a** MTT assays were performed to determine the cell viability of cardiomyocytes transfected with *ZFAS1-P*, with and without pretreatment with *BAPTA*. **P < 0.01 vs. control group, ^##^*P* *<* 0.01 vs. *ZFAS1-P*, *n* = 15. **b** LIVE/DEAD Viability/Cytotoxicity Kit stains were used to detect the viability of *ZFAS1*-overexpressing cardiomyocytes, with or without pretreatment with *BAPTA*. **P < 0.01 vs. control group, ^##^*P* *<* 0.01 vs*. ZFAS1-P*, *n* = 5. Magnification, ×200. **c**–**e** Western-blot analysis of the expression of apoptosis related proteins. **P < 0.01 vs. control group, ^##^*P* < 0.01 vs*. ZFAS1-P*, *n* = 4–5. **f** MTT assays were performed to determine the effects of *BAPTA* on the cell viability of *ZFAS1*-overexpressing cardiomyocytes. *P < 0.05 vs. control group, ^#^*P* < 0.05 vs. *ZFAS1-*FD, *n* = 10. **g**, **h** LIVE/DEAD Viability/Cytotoxicity Kit Stains were used to determine the viability of cardiomyocytes transfected with *ZFAS1-*FD, with or without pretreatment with *BAPTA*. **P < 0.01 vs. control group, ^##^*P* < 0.01 vs. *ZFAS1-*FD, the results are expressed as the means ± SEM of three independent experiments^.^ Magnification, ×200. **i** Effects of BAPTA on the mitochondrial membrane potential of *ZFAS1-*overexpressing cardiomyocytes were determined by JC-1 staining. Magnification, ×1200. Similar results were consistently observed in another two batches of cells. **j**, **k** *ZFAS1*-FD-induced cardiomyocyte apoptosis and BAPTA pretreatment could rescue its effects, which was validated by TUNEL assay. Blue, DAPI staining for nucleus; green, TUNEL-positive staining for apoptotic cells. *P *<* 0.05 vs. control group, ^#^*P* *<* 0.05 vs. *ZFAS1-*FD, the results are expressed as the means ± SEM of three independent experiments. Magnification, ×200. **l–n** Western-blot analysis of the expression of apoptosis related proteins. *ZFAS1-*FD increased the expression of Bax, caspase-3, and caspase-9, and *BAPTA* pretreatment could suppress this change.*P < 0.05, **P < 0.01 vs. control group, ^#^*P* < 0.05, ^##^*P* *<* 0.01 vs. *ZFAS1-*FD, *n* = 4 for caspase-3 and caspase-9, *n* = 5 for Bax. Data are presented as means ± SEM.

It has been reported that *BAPTA* (ethane-N,N,N′,N′-tetraacetic acid) is a calcium chelator that resists cytosolic Ca^2+^ overload^25^. Our previous study had verified that *ZFAS1* could induce cytosolic Ca^2+^ overload^18^. At present, forced expression of *ZFAS1* with transfection of *ZFAS1*-P directly caused injured cell viability in non-hypoxic NMCMs. These effects of *ZFAS1* were effectively reversed by pre-treatment with *BAPTA* (Fig. 5a, b). At the molecular level, BAPTA inhibited the *ZFAS1* overexpression-mediated upregulation of pro-apoptotic proteins Bax, caspase-3, and capase-9 (Fig. 5c–e). Our previous study identified a functional domain of *ZFAS1* named *ZFAS1*-FD, which showed similar effects on modulation of SERCA2a via direct binding. Similarly, *ZFAS1*-FD caused decreased cell viability, compromised mitochondrial membrane potential and increased apoptosis of cardiomyocytes, which could been ameliorated by BAPTA (Fig. 5f–k). Furthermore, compared with the *ZFAS1*-FD overexpressed NMCMs, the expression of apoptosis-associated proteins caspase-3, caspase-9, and Bax were significantly decreased in the BAPTA-treated NMCMs (Fig. 5l–n).

## Discussion

This study presents the following significant results: (1) knockdown of *ZFAS1* protects cardiomyocytes against MI and hypoxia treatment-induced apoptosis; (2) upregulation of *ZFAS1* expression induces cardiomyocyte apoptosis; (3) *ZFAS1* induces cardiomyocyte apoptosis by inhibiting SERCA2a and causing cytosolic Ca^2+^ overload.

To further explore the role of *ZFAS1* on the heart, *ZFAS1* cardiac-specific knock-in mice were constructed. Consistent with our previous study, *ZFAS1* knock-in mice (TG) showed impaired cardiac function, deleteriously altered Ca^2+^ homeostasis, and repressed expression of SERCA2a. At the subcellular level, *ZFAS1* induced mitochondrial swelling and markedly decreased the mitochondrial membrane potential. At the molecular level, *ZFAS1* activated the mitochondria apoptosis pathway, which could be nearly abolished by a calcium chelator. The effects of *ZFAS1* were readily reversible upon knockdown of this lncRNA. Notably, *ZFAS1-FD* mimicked the effects of the full-length *ZFAS1* in regulation of cardiomyocyte apoptosis. Based upon these findings, we concluded that *ZFAS1*, an endogenous SERCA2a inhibitor, induces mitochondria-mediated apoptosis by causing cytosolic Ca^2+^ overload. Therefore, anti-*ZFAS1* might be considered as a new therapeutic strategy for protecting cardiomyocytes from MI-induced apoptosis.

In the past few years, we have devoted ourselves to the study of lncRNA-*ZFAS1* in cardiovascular diseases. By collecting and detecting the blood samples from AMI patients, non-AMI control subjects and healthy volunteers, we identified that *ZFAS1* was a potential biomarker for MI^17^. Our recently published study suggested that lncRNA-*ZFAS1* is a SERCA2a inhibitor that causes intracellular Ca^2+^ overload and contractile dysfunction in a mouse model of MI^18^. In this study, we further demonstrated the inhibitory effect of *ZFAS1* on SERCA2a by using cardiac-specific *ZFAS1* knock-in mice. During the course of this study, a research article on *ZFAS1* in the heart was published, wherein the authors demonstrated that *ZFAS1* promotes apoptosis by acting as a ceRNA to reduce the functional availability of miR-150 and increase the level of C-reactive protein^26^. As a key regulatory factor of MI, we believe that the regulation mechanism of *ZFAS1* is necessarily diverse. The finding in the present study that *ZFAS1* caused intracellular Ca^2+^ overload, thereby inducing cardiomyocyte apoptosis is another alternative mechanism for its pro-apoptotic function.

In fact, there was a limitation of our present study. In the cardiac tissue of TG, the mRNA level of SERCA2a was decreased. To verify the question how *ZFAS1* altered the mRNA level of SERCA2a, we performed the bioinformatic prediction. By computer blasting and RNAfold website calculating, we found that *ZFAS1* could bind to the mRNA of SERCA2a both at the CDS region (a total of 94 base sequences from 413 to 507 in *ZFAS1* are complementary to 93 sequences from 1990 to 2083 in SERCA2a CDS region) and the 3′UTR (a total of 178 base sequences from 564 to 742 in *ZFAS1* are complementary to 165 sequences from 24 to 189 in SERCA2a 3′UTR region) with lower free energy. The results above indicated that *ZFAS1* might interact with mRNAs through direct antisense complementary to induce degradation of the SERCA2a mRNA. However, it is lack of rigorous studies to elucidate the exact mechanisms. We believed that mechanism of lncRNAs regulating mRNAs was worthy of further study.

The findings in the present study, the published studies by our laboratory and other research group indicated that lncRNA-*ZFAS1* plays important roles in the setting of MI. By inhibiting SERCA2a, *ZFAS1* not only caused cardiac dysfunction but also cardiomyocytes apoptosis during MI. Particularly, knockdown of *ZFAS1* showed protective effects on the ischemia myocardium or hypoxia NMCMs This study strengthened the evidence that *ZFAS1* might be a potential target for the treatment of MI.

## Supplementary information

Supplementary Figure S1

Supplementary Figure S2

Supplementary Figure S3

Supplementary Figure S4

Supplementary Figure S5

Supplementary Figure S6

Supplementary Figure S7

Supplementary Figure S8

Supplementary Figure S9

Supplementary Figure S10
