# Supplementary figures and images for: lncRNA-ZFAS1 induces mitochondria-mediated apoptosis by causing cytosolic Ca2+ overload in myocardial infarction mice model
Source: Cell Death Dis. 2019 Dec 9;10(12):942. doi: 10.1038/s41419-019-2136-6 (PMC6901475; doi:10.1038/s41419-019-2136-6)

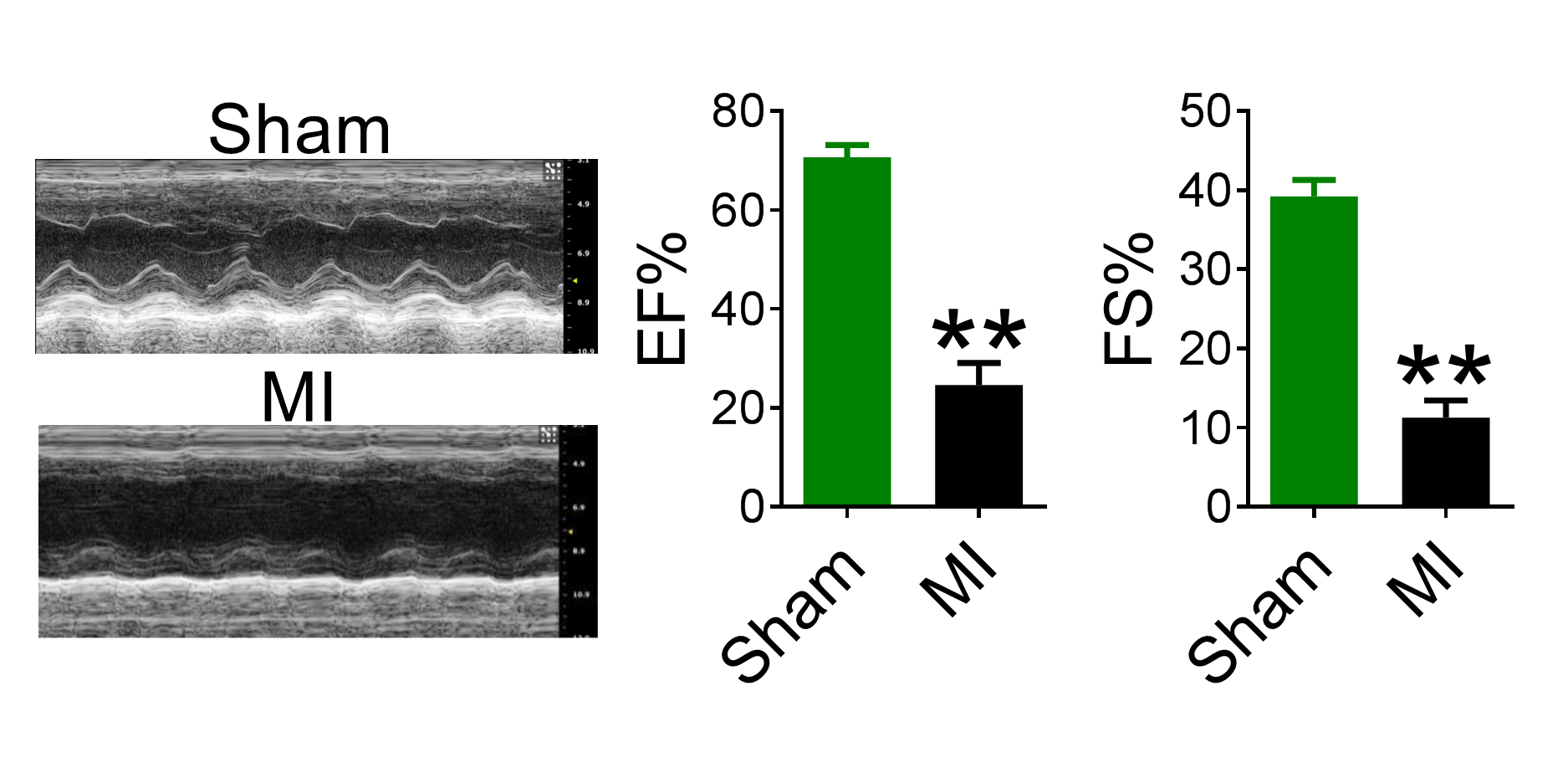

Supplement: Supplementary file 1 — Supplementary Figure S1 [file 41419_2019_2136_MOESM1_ESM.tif]

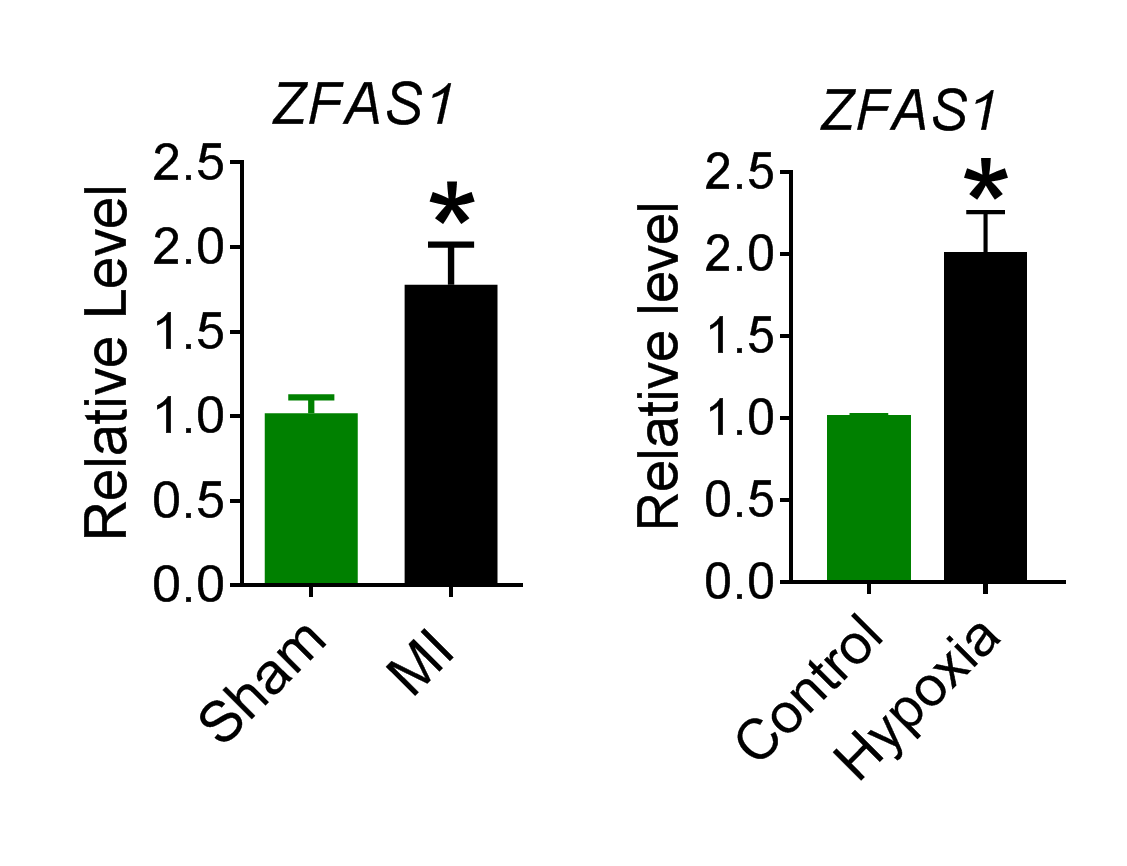

Supplement: Supplementary file 2 — Supplementary Figure S2 [file 41419_2019_2136_MOESM2_ESM.tif]

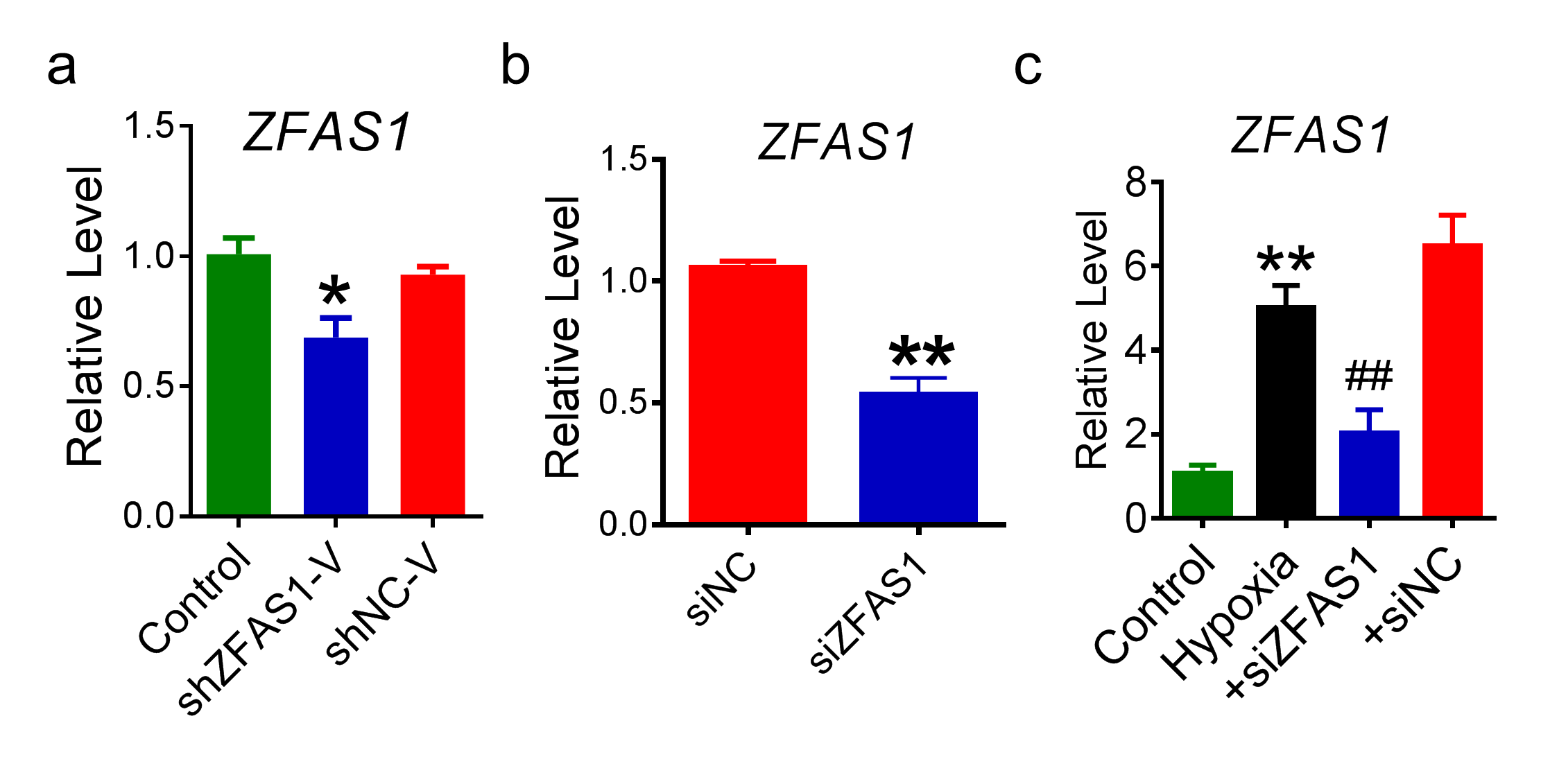

Supplement: Supplementary file 3 — Supplementary Figure S3 [file 41419_2019_2136_MOESM3_ESM.tif]

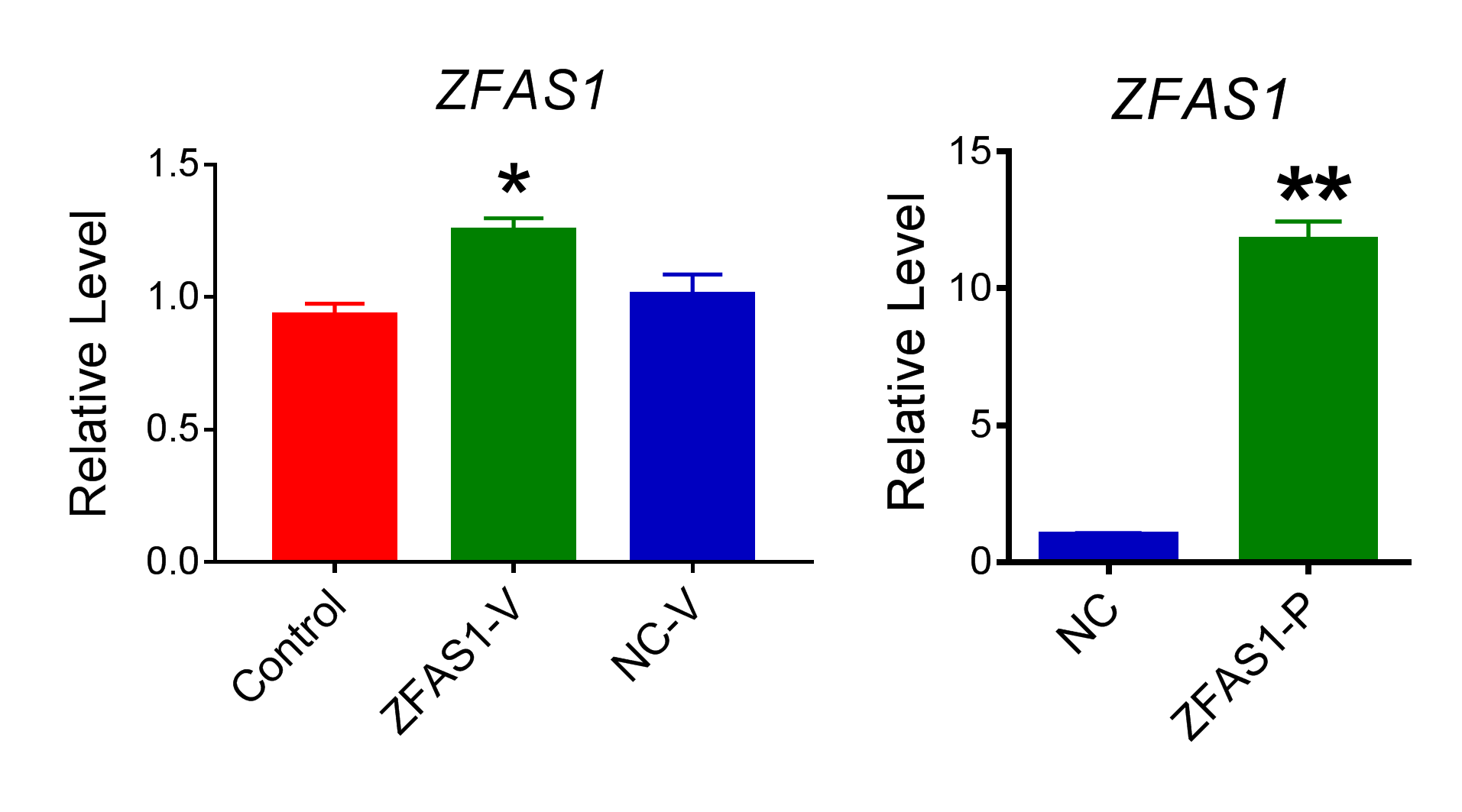

Supplement: Supplementary file 4 — Supplementary Figure S4 [file 41419_2019_2136_MOESM4_ESM.tif]

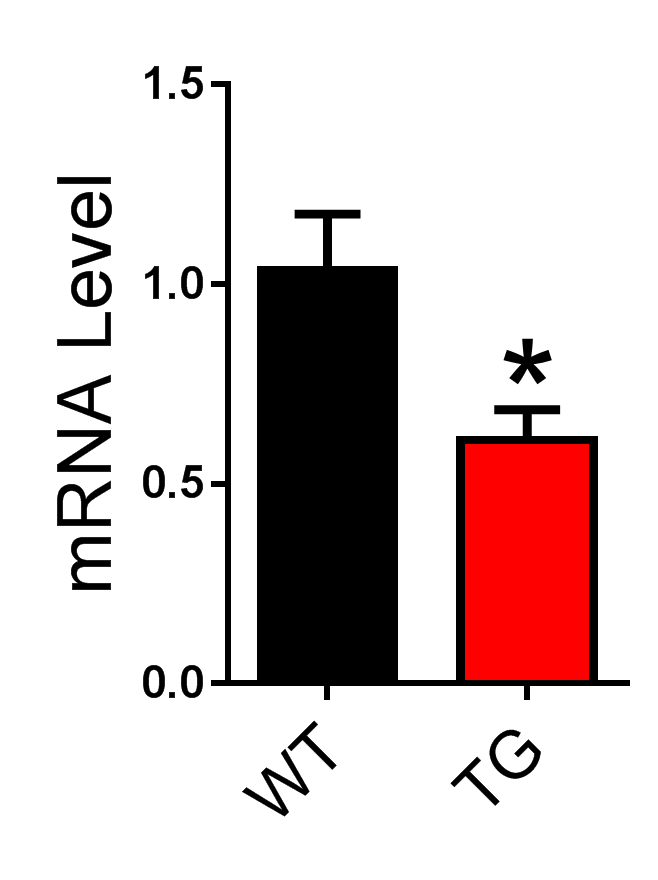

Supplement: Supplementary file 5 — Supplementary Figure S5 [file 41419_2019_2136_MOESM5_ESM.tif]

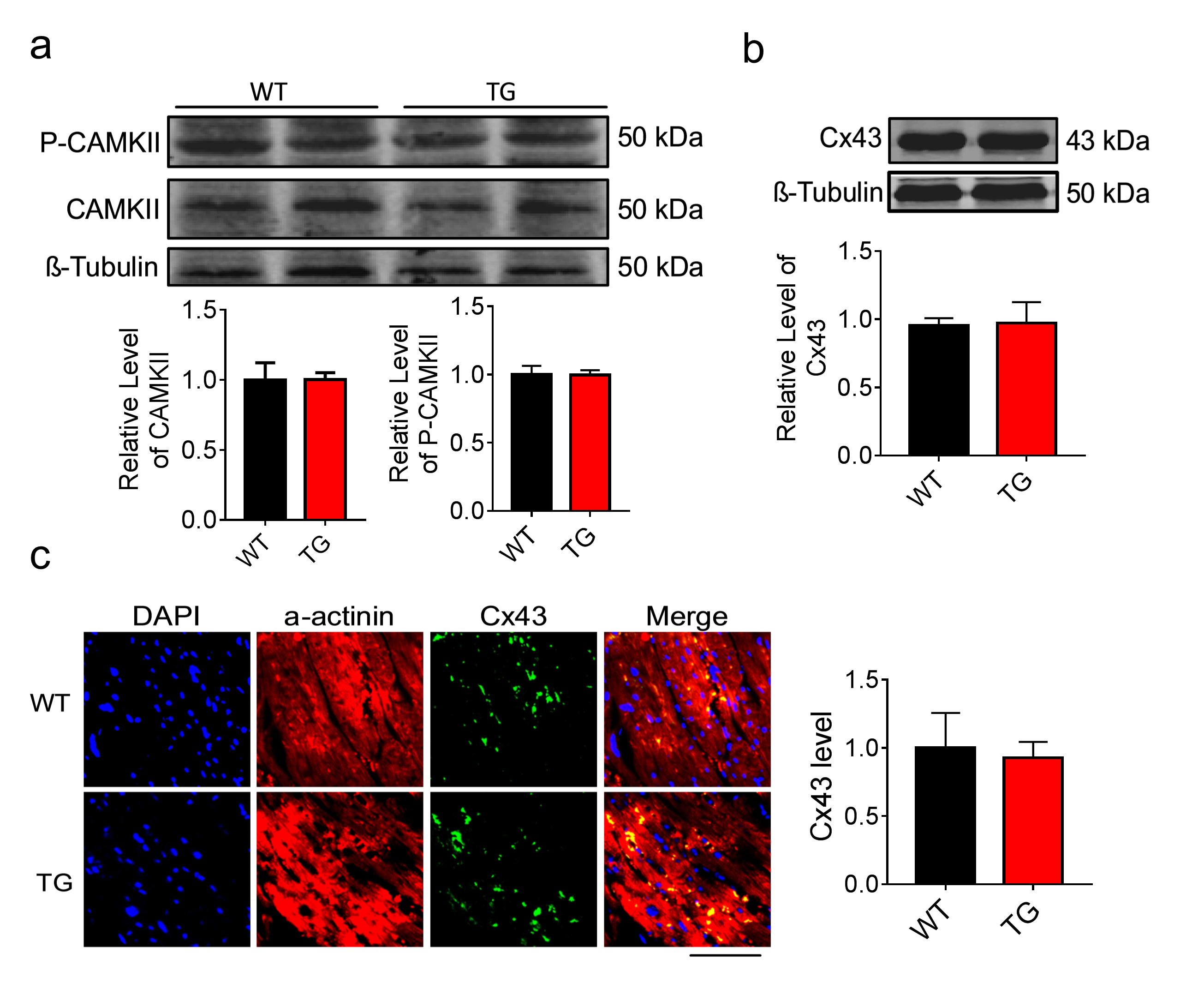

Supplement: Supplementary file 6 — Supplementary Figure S6 [file 41419_2019_2136_MOESM6_ESM.tif]

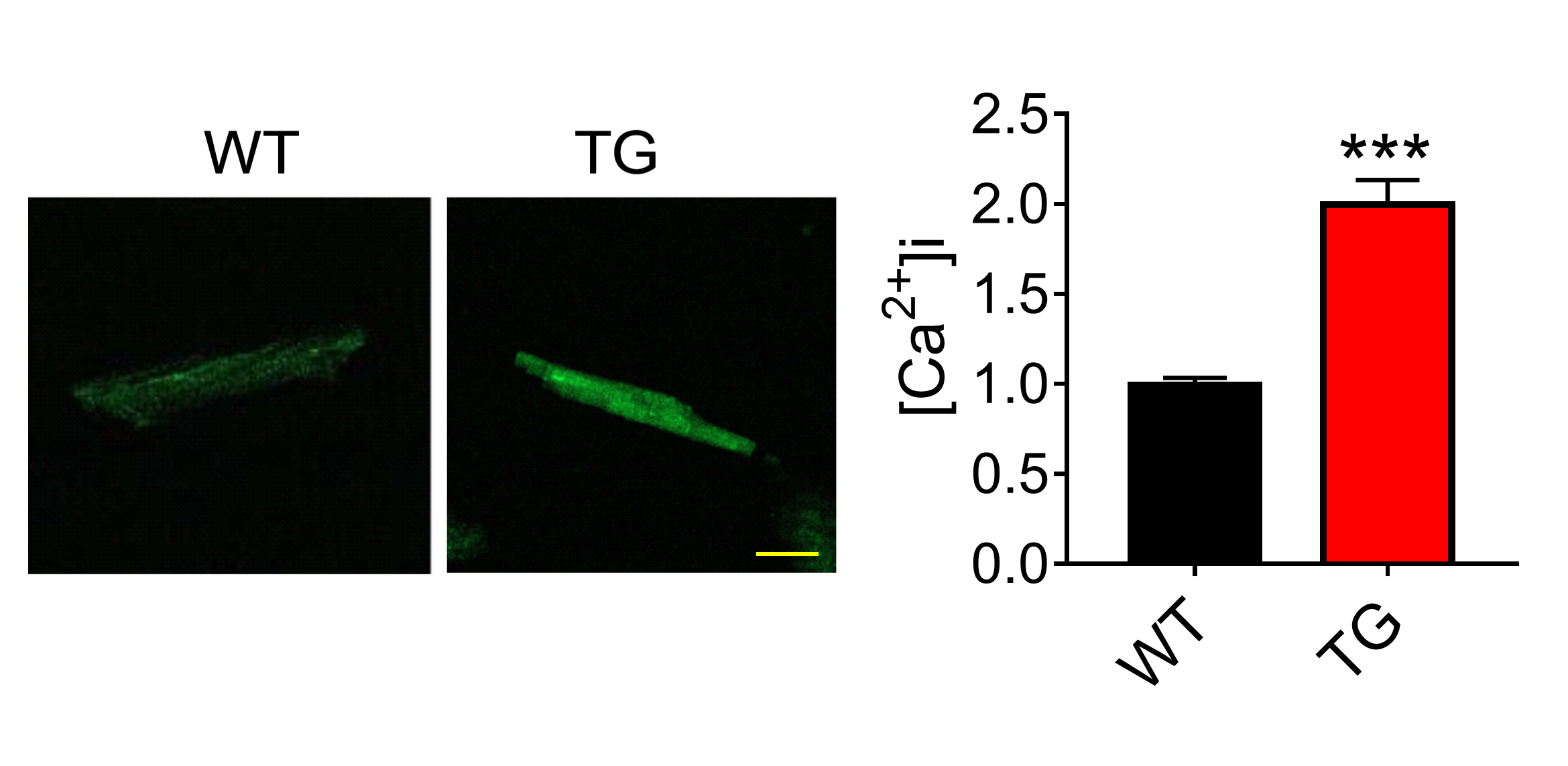

Supplement: Supplementary file 7 — Supplementary Figure S7 [file 41419_2019_2136_MOESM7_ESM.tif]

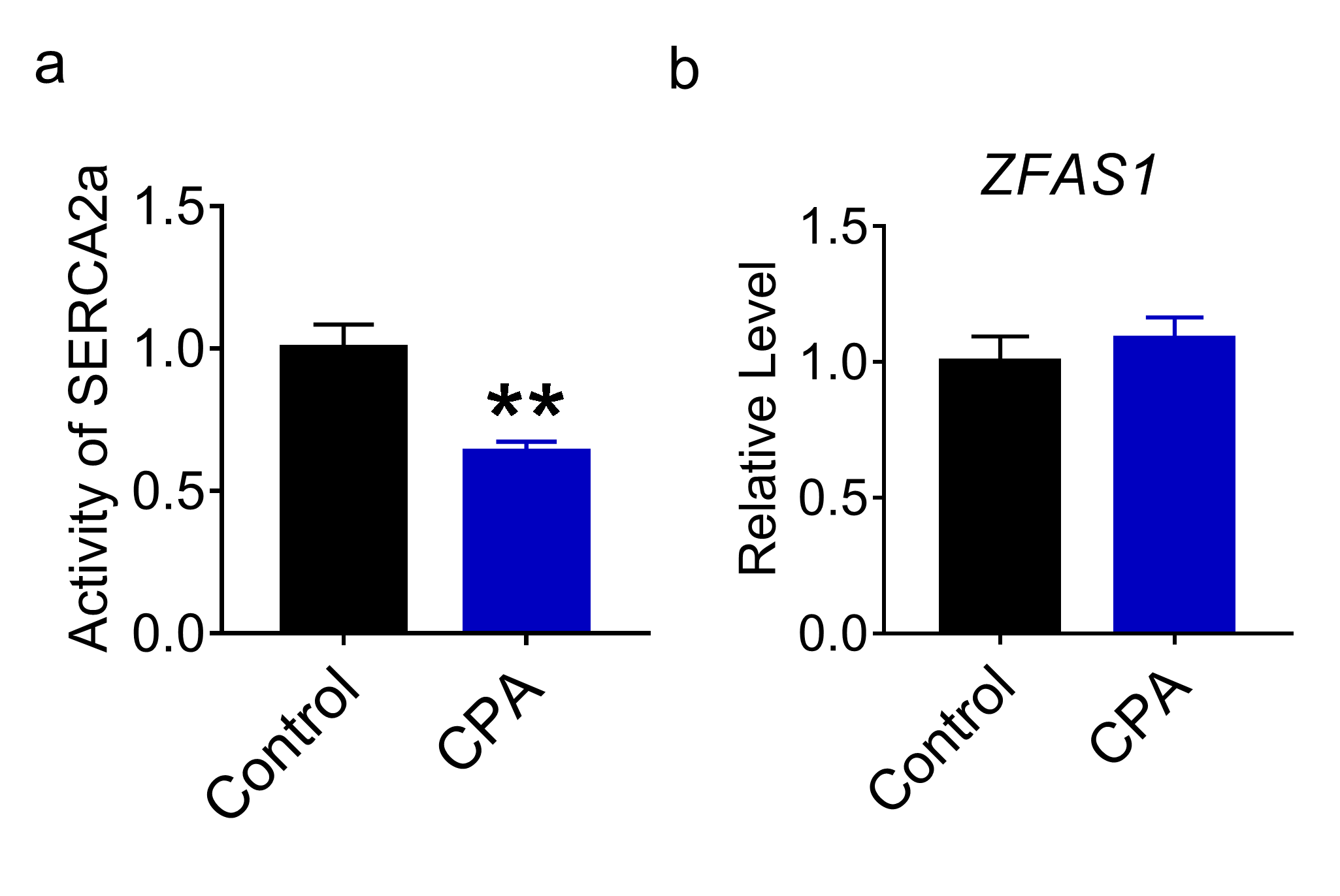

Supplement: Supplementary file 8 — Supplementary Figure S8 [file 41419_2019_2136_MOESM8_ESM.tif]

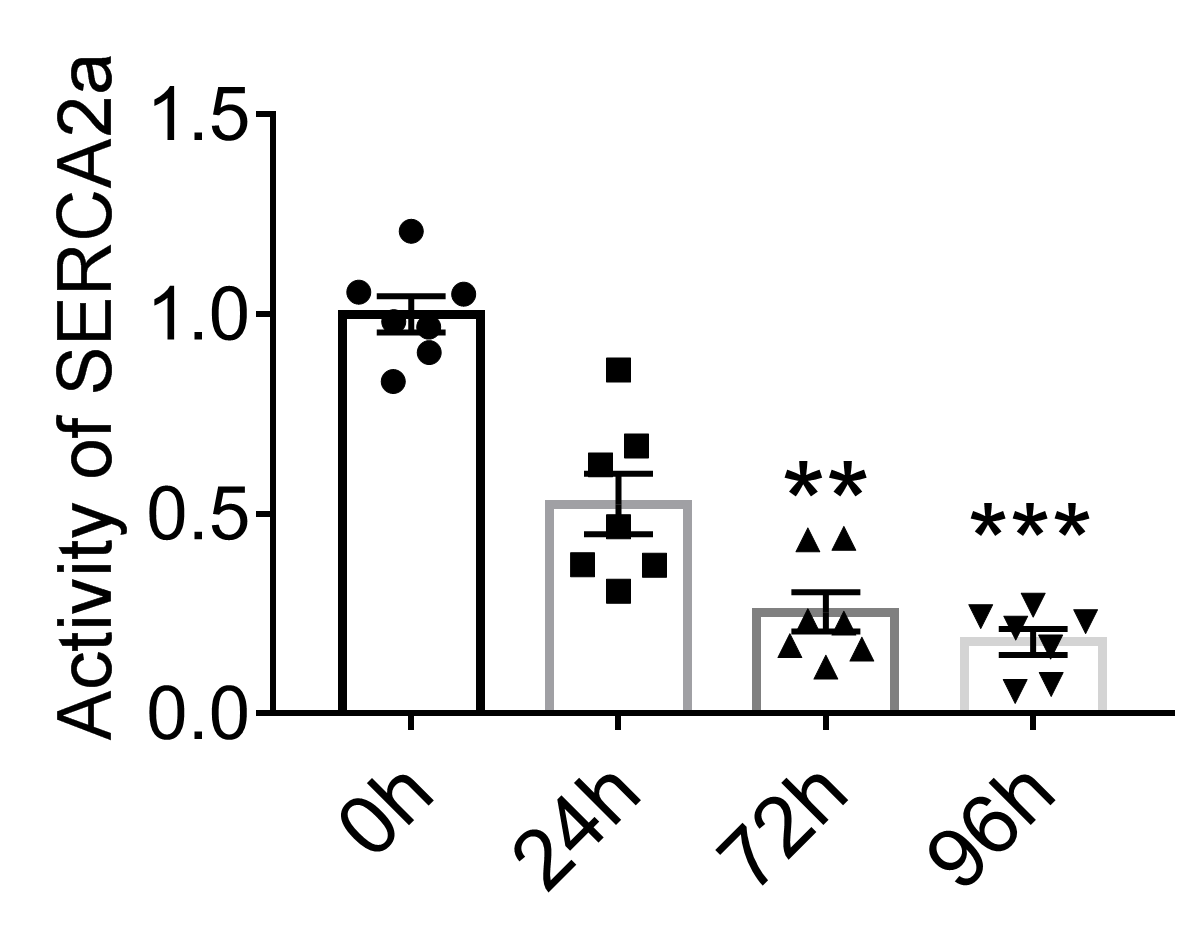

Supplement: Supplementary file 9 — Supplementary Figure S9 [file 41419_2019_2136_MOESM9_ESM.tif]

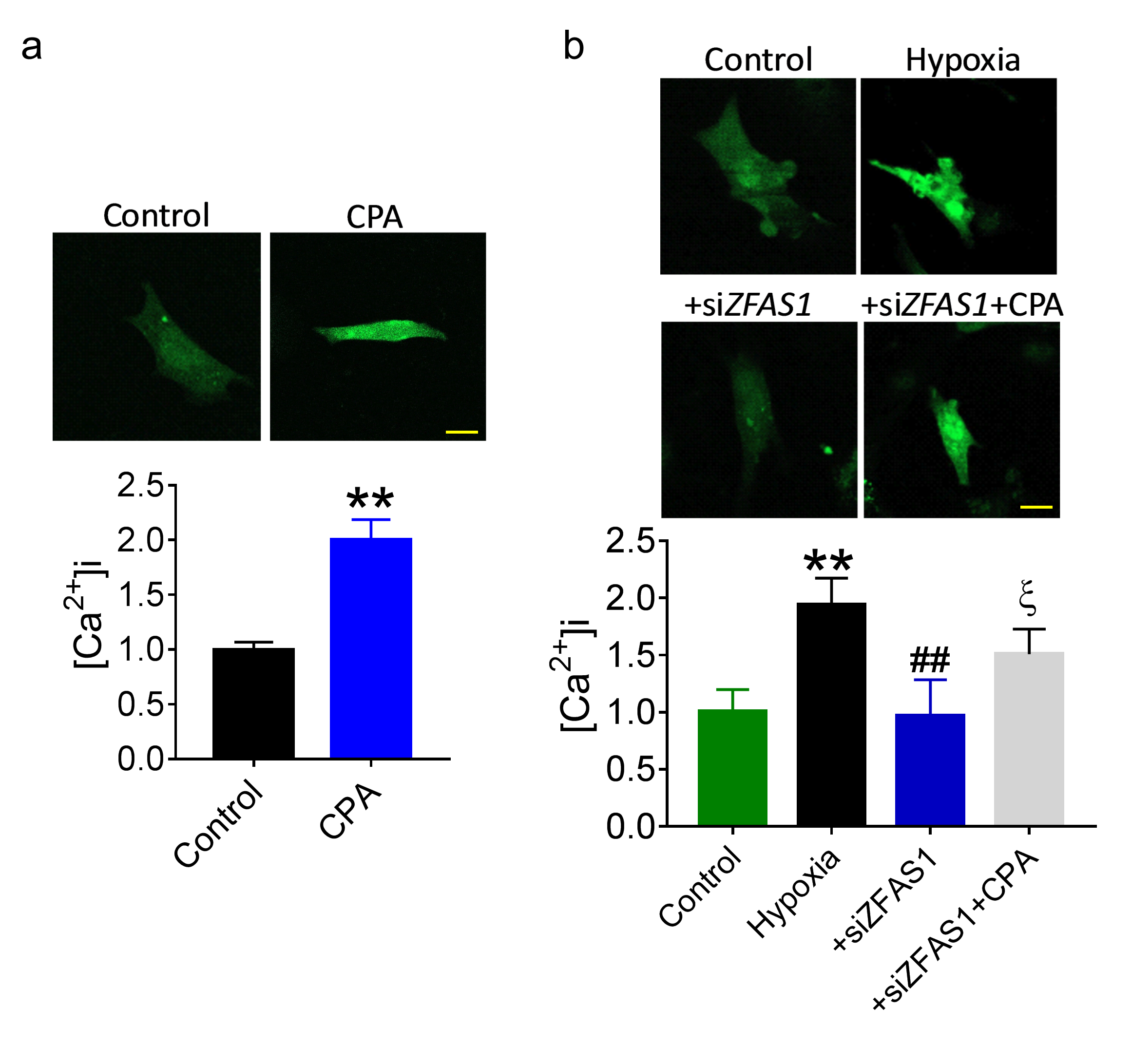

Supplement: Supplementary file 10 — Supplementary Figure S10 [file 41419_2019_2136_MOESM10_ESM.tif]
